# Impact of Melatonin in Solid Organ Transplantation—Is It Time for Clinical Trials? A Comprehensive Review

**DOI:** 10.3390/ijms19113509

**Published:** 2018-11-08

**Authors:** Philipp Stiegler, Augustinas Bausys, Bettina Leber, Kestutis Strupas, Peter Schemmer

**Affiliations:** 1 Department General, Visceral and Transplant Surgery, Medical University of Graz, Graz 8036, Austria; philipp.stiegler@medunigraz.at (P.S.); abpelikanas@gmail.com (A.B.); 2 Transplant Center Graz, Medical University of Graz, Graz 8036, Austria; bettina.leber@medunigraz.at; 3 Faculty of Medicine, Vilnius University, Vilnius 03101, Lithuania; kestutis.strupas@mf.vu.lt; 4 Department of Abdominal Surgery and Oncology, National Cancer Institute, Vilnius 08660, Lithuania

**Keywords:** heart transplantation, lung transplantation, liver transplantation, kidney transplantation, pancreas transplantation, melatonin, review

## Abstract

Solid organ transplantation is the “gold standard” for patients with end-stage organ disease. However, the supply of donor organs is critical, with an increased organ shortage over the last few years resulting in a significant mortality of patients on waiting lists. New strategies to overcome the shortage of organs are urgently needed. Some experimental studies focus on melatonin to improve the donor pool and to protect the graft; however, current research has not reached the clinical level. Therefore, this review provides a comprehensive overview of the data available, indicating that clinical evaluation is warranted.

## 1. Introduction

For some end-stage organ diseases, solid organ transplantation is the only potentially curative treatment option. In end-stage renal failure, kidney transplantation (KTx) is considered as a better treatment option than hemodialysis, due to the improved quality of life and cost-effectiveness [1]. Similarly, pancreas transplantation (PTx) also improves quality of life as a result of the patient’s independence from insulin injections, glucose level monitoring, and awareness of fasting hypoglycaemia [2]. In recent decades, the transplantation field has dramatically increased and now many organs, including the uterus, small bowel, penis, face or limbs, are available for transplantation under routine or experimental settings [3,4,5,6,7,8]. According to the database of the Global Observatory on Donation and Transplantation, more than 107000 solid organs were transplanted in 2016 [9]. Despite the increasing variety of organs available for transplantation, the heart, lung, liver, pancreas, and kidneys remain the most frequently transplanted organs, accounting for more than 99% of all solid organ transplantations. Demand for these organs is constantly increasing, but organ supplies remain insufficient to meet the demand [10,11], leading to an increased organ shortage, which some authors consider as the greatest challenge in modern transplant medicine [11,12,13]. Therefore, strategies to expand the donor pool are mandatory. The use of organs from extended criteria donors (ECD) may help to overcome the organ shortage [10]. However, marginal grafts have to be carefully evaluated and are known to be susceptible to ischemia/reperfusion injury (IRI) [14]. IRI is one of the major risk factors for unfavorable outcomes after transplantation resulting in short- and long-term complications, such as delayed graft function and acute or chronic rejection [15]. To reduce IRI, the development of novel therapeutic approaches is crucial for further progress of transplantation medicine and the use of ECD organs. Various strategies, which include donor preconditioning [16,17,18], the optimization of organ perfusion and storing conditions [19,20], as well as the development of drugs targeting IRI, are under investigation [21]. Melatonin (MLT), the main secretory product of the pineal gland, has many biological activities, including immunoregulatory and anti-oxidative effects [22,23,24]. The direct free radical scavenging activity of MLT was first reported more than 25 years ago, in the early 90s [25]. Later, some studies also showed the indirect anti-oxidative properties of this substance. The multiple working pathways of MLT include, among others, the stimulation of anti-oxidative enzymes, the upregulation of glutathione synthesis, the neutralization of nitrogen-based toxicants, and the suppression of pro-oxidative enzymes activity [26,27,28,29,30]. However, for a long time, it was considered as a strict immune-stimulator and did not attract the attention of the transplantation community. Growing knowledge revealed that MLT has various effects on the immune system [31,32], including some immune-suppressive features [33]. Moreover, MLT toxicity itself is extremely low, as shown in animal [34,35] as well as human studies [34,36,37]. Until now, MLT has been tested in more than ten preclinical studies on solid organ transplantation (Table 1) with controversial results. The potentially beneficial effect of MLT in the whole, broad field of transplantation—also including bone, ear, ovarian, and testicular transplantation—was addressed by Esteban-Zubero et al. in 2016 [38]. However, the data available are still focusing on basic research, without being directly applicable in clinical settings. This comprehensive review provides an overview of the current evidence on the role of MLT in solid organ transplantation.

## 2. Comprehensive Review

### 2.1. Heart Transplantation (HTx)

The era of HTx began in 1967, when the first transplantation was performed by Christiaan Bernard in South Africa. Since those days, significant progress has been achieved, reaching one-year survival rates of 87%. The median survival exceeds ten years, and the expected quality of life is excellent [39,40]. Currently, HTx is considered as the gold standard therapy for patients with end-stage heart failure. Unfortunately, the shortage of suitable standard criteria donor hearts is growing [41]. Therefore, the use of organs from ECD is dramatically increasing [42], although marginal grafts are very susceptible to IRI. Therefore, the need for anti-IRI strategies in HTx is urgent.

Many experimental studies have showed the protective effects of MLT against myocardial IRI in a non-transplant setting [43,44,45,46,47]. Sihai and colleagues [48] investigated MLT in transplantation settings as an additive substance to preservation solutions. Explanted rat hearts were stored for 12 hours in a St. Thomas solution or the same solution supplemented with 100 µmol/L of MLT. After reperfusion, the aortic flow, cardiac output, systolic pressure, left ventricle minute work, and coronary flow was significantly improved with MLT. Moreover, creatine kinase release was reduced and high-energy phosphates (phosphocreatine and β-adenosine triphosphate) were preserved much better in hearts stored in solutions supplemented with MLT. An ultrastructure analysis using electron microscopy supported these findings. After 12 h of static cold storage, mitochondria injury was blunted and glycogen granules were well preserved when MLT was applied. This study did not show the exact working mechanisms of MLT but it indicated that anti-oxidative properties against IRI are involved. In contrast, 200 mg of MLT administered intravenously and intracoronarily did not reduce myocardial IRI in a porcine closed-chest ischemia-reperfusion model [49]. Moreover, MLT was tested against cardiac IRI in non-transplant clinical trials and the results are also ambiguous. The administration of MLT during primary percutaneous coronary intervention in patients with ST-elevation myocardial infarction did not improve the myocardial salvage index [50]. On the other hand, some cardioprotective effect of MLT was shown during coronary artery bypass surgery [51] and major vascular surgery [52].

However, these differences may be related to methodological differences of the studies. In both clinical trials, which confirmed the cardioprotective effects of MLT, the substance was administered not only intravenously but also orally. Administration time and route can limit MLT application in many clinical situations, but not in transplant surgery. There is no limitation for MLT administration before organ retrieval, during storage, at any stage of implantation, or during the postoperative course.

In HTx, the anti-IRI effect is not the only potentially beneficial effect of MLT. Jung et al. [33] hypothesized that MLT can mediate immunosuppressive effects following alloantigen exposure in a dose-dependent manner and tested this hypothesis in a rat model of acute cardiac allograft rejection. Study results showed significantly reduced lymphocyte proliferative capacity, abrogated acute allograft rejection and prolonged graft survival in animals who were treated with a high dose of MLT. These results were reproduced by Liu et al. [53], who found that MLT improved graft survival and also alleviated the inflammatory response and apoptosis in cardiac tissue after HTx.

Taking the current knowledge together, MLT application in HTx seems to be promising when MLT is added to preservation solutions. Moreover, some evidence exists about the immunosuppressive properties of MLT. However, not all the experimental and clinical studies support the benefits of MLT and it remains unclear if the route of application can influence the substance efficacy. 

### 2.2. Lung Transplantation (LuTx)

Organ shortage is significant in all transplant medicine, but it is even more acute in LuTx [64] due to the high incidence of lung injury in potential deceased donors [64] and the fact that living donor transplantation is extremely complicated [65]. After transplantation, up to 30% of recipients develop primary graft dysfunction within the first 72 h after reperfusion [66], which is mainly caused by pulmonary IRI leading to significant postoperative morbidity and mortality [67].

The effect of MLT on post-transplant lung IRI after prolonged ischemia was studied by Inci and colleagues [54]. In this experimental study, both donor and recipient rats were treated with intraperitoneal injections of MLT before organ retrieval and reperfusion, respectively. The results of the study showed that treatment reduced lipid peroxidation and myeloperoxidase activity in lung tissue and nitrite levels in bronchoalveolar lavage and functional parameters improved. In contrast, an experimental study performed by Santana-Rodriguez failed to show the beneficial effects of MLT against IRI and the acute rejection of lung grafts [55]. However, many methodological differences between these two studies should also be taken into consideration. In first study, MLT was administered to both donor and recipient intraperitoneally, while in the second, only the recipient received a single dose of MLT subcutaneously. Another difference was the organ storage time before transplantation in the first study, with 18 h of cold static storage more likely mimicking the situation of ECD. In the second study, the lungs were transplanted after 6 h of storage, and therefore organ quality at the time of reperfusion was considerably better. Taken together, it is not clear if the effectiveness of MLT depends on the route and time of administration, if MLT is effective in lung transplantation in general or only for marginal quality organs, and also if MLT is effective only against very early IRI or positively influences long-term outcomes too. 

Generally, some evidence about the beneficial effect of MLT against IRI in lung grafts exists. However, there is no answer as to whether MLT could be beneficial in every LuTx or only in the case of ECD. Moreover, the optimal dose and route of administration need to be clarified.

### 2.3. Liver Transplantation (LTx)

The number of patients who are candidates for LTx is steadily increasing, thus a discrepancy between the numbers of patients on the waiting list and the available organs is augmenting [68]. New strategies to overcome liver shortages are coming to clinical practice. They include the use of ECD organs, living donor LTx, and the consideration to implement systematic splitting graft procedures in cadaveric organs [68]. However, marginal quality livers are under higher risk of IRI [69] and strategies to overcome IRI should be investigated. As mentioned above, living donor transplantation is an option for reducing organ shortages in LTx. The prevalence of this procedure is increasing, especially in Asian countries [70].

Since the use and safety of marginal quality livers need to be increased, Zaouali and colleagues investigated MLT as an additive to preservation solutions targeting IRI in steatotic liver grafts [56]. Livers preserved in solutions containing MLT showed reduced vascular resistance. Authors demonstrated that the effect of MLT correlated with the generation of nitric oxide, the prevention of oxidative stress, and inflammatory cytokine release. The beneficial effect of MLT was observed not only in steatotic but also healthy livers. Kireev and colleagues [71] also investigated the effect of MLT on IRI in fatty livers but used a non-transplant experimental protocol and a different route of MLT administration through ligation of the portal vein and the hepatic artery. Treatment with MLT via several routes and at several time points was tested. The results of this study showed that MLT protected steatotic livers against IRI in different ways, and better when administered orally. The burden of oxidative stress was mitigated by the scavenging capacity provided by MLT itself and by the activation of antioxidant enzymes. Moreover, MLT improved the recovery of mitochondrial dysfunction and the hepatocyte ability to produce ATP, and downregulated the expression of pro-apoptotic genes. Hepatoprotective effects of the substance were also observed in biochemical blood parameters and histological liver samples. The increase in aminotransferases after IRI was blunted when MLT was applied. Liang et al. [72] also tested MLT in a non-transplant experimental protocol of warm ischemia followed by reperfusion and resection of non-ischemic liver lobes. The authors demonstrated a clear hepatoprotective effect of MLT by showing significantly improved animal survival, decreased transaminase levels, indices for necrosis, liver damage, leukocyte infiltration, and iNOS expression. MLT application also resulted in the decreased expression of IKKα, JNK1, and cJUN and reduced the expression of proliferating cell nuclear antigen (PCNA) and Ki67. The authors concluded that MLT is hepatoprotective most likely via mechanisms including the inhibition of IkappaB kinase (IKK) and c-Jun N-terminal kinase (JNK) pathways and the regulation of cell proliferation.

Another study focusing on the role of MLT in LTx was published in 2005 by Vairetti et al. [57]. This experimental study examined whether MLT acts on bile flow and IRI, caused by cold storage and reperfusion in an isolated rat liver model. After liver explantation, Krebs-Henseleit bicarbonate (KHB) buffer with or without MLT was used for organ storage. The study results demonstrated the beneficial effect of MLT on bile flow production, bile bilirubin excretion, and liver tissue ATP level preservation. Later, the same group performed an additional histochemical analysis of the liver grafts and demonstrated that MLT improved mitochondria function [73]. 

Moussavian and colleagues [58] published results from an isolated rat liver perfusion model using preconditioning with a multidrug cocktail consisting of curcumin, simvastatin, *N*-acetylcysteine, erythropoietin, pentoxyphylline, MLT, glycine, and methylprednisolone. A different multidrug cocktail composition (pentoxyphylline, glycine, deferoxamine, *N*-acetylcysteine, erythropoietin, MLT, and simvastatin) was published by Heesen and colleagues [59]. Both studies showed positive effects of preconditioning but since the drug cocktails used consisted of various substances, it is impossible to interpret the role of MLT.

Another group used MLT in combination with trimetazidine as an additive targeting IRI for preservation solutions in an isolated fatty liver rat model [60]. The authors concluded that the supplement used in this study improved steatotic liver preservation through 5′ AMP-activated protein kinase activation, which in turn circumvents endoplasmic reticulum stress and modulates liver autophagy.

A very recent study investigated the potential of MLT to improve the regeneration of hepatic grafts exposed to ischemia in a mouse model [61]. MLT was administered intraperitoneally before the operation and after reperfusion in models of IRI and partial hepatectomy (IRI + PH). For the small for size liver transplantation model (SFS–LT), MLT was used as a supplement for perfusion and the storing solution. Additionally, MLT was injected intraperitoneally to the recipient before transplantation and immediately after reperfusion. The results of this study showed that in the IRI+PH model, MLT reduced liver injury, enhanced liver regeneration, and promoted interleukin (IL6, IL10, TNF-α) release from inflammatory monocytes. The MLT-induced increase of IL6 significantly improved hepatic microcirculation and the survival of the experimental animals. In the SFS–LT model, MLT promoted graft regeneration and increased recipient survival.

MLT was already tested in a clinical non-transplant liver surgery setting [74]. Treatment with MLT reduced postoperative transaminase levels, but the results were not statistically significant.

Altogether, the possible role of MLT in LTx has been widely studied. The benefits of MLT against IRI have been shown in several experimental animal models, including studies mimicking healthy and steatotic livers. The recent living donor liver transplantation model in mice also showed that MLT may ameliorate IRI in SFS liver and even restore regeneration. In addition, the safety of MLT application during liver surgery was also confirmed. On the other hand, only rodent models were used for experimental studies and there is a lack of large animal studies which would precisely mimic LTx in humans and would evaluate substance influence for long-term results after transplantation. Nonetheless, the role of MLT in LTx has been studied better than for other organ transplantations. Therefore, we consider that the application of MLT in clinical trials should start focusing on LTx.

### 2.4. Kidney Transplantation (KTx)

The kidney is the most frequently transplanted solid organ [75]. Various factors can influence the outcome of transplanted kidneys, but IRI is the major cause of delayed graft function, also associated with graft rejection and chronic graft dysfunction [76,77]. IRI may occur in a kidney transplanted from a living donor but it is much more common and severe in kidneys from deceased donors [78]. Since the majority of kidneys for transplantation still come from cadaveric donors [79], IRI remains a hot topic in the renal transplantation field.

MLT has been shown to protect kidneys against IRI in several experimental studies [80,81,82,83]. However, most of these studies examined warm ischemia and reperfusion, therefore MLT effectiveness in KTx remained unclear until Li and colleagues [62] tested this substance in an experimental KTx model. The results of this study showed that donor preconditioning with MLT improved recipient survival and decreased blood urea nitrogen, creatinine, transaminases, and lactate dehydrogenase levels in plasma after transplantation by up to 71%. Moreover, MLT significantly reduced the histological index of tubular damage, induced the enzymatic activity of superoxide dismutase, and reduced the activity of lipid hydroperoxide in kidneys. At the same time, the expression of NF-κB p65, iNOS, and caspase-3 were down-regulated. The authors concluded that donor preconditioning with MLT protected grafts from IRI-induced renal dysfunction and tubular injury most likely through its anti-oxidative, anti-apoptotic, and NF-κB inhibitory capacity.

While the kidney is the most frequently transplanted organ, it is surprising that only one experimental study has investigated MLT as a potential agent against IRI in the KTx model. Although the benefit of donor preconditioning with MLT could be shown, the lack of studies limits the translation into clinical trials.

### 2.5. Pancreas Transplantation (PTx)

PTx is the main method to restore physiological glycaemic control and prevent the progression of complications in diabetic patients [75]. Incredible progress has been made in this field in the last decades; for example, one-year graft survival rates increased from 20% in the early 80s to more than 80% nowadays [84]. This progress is mainly thanks to improved surgical techniques, better immunosuppressive regimens, and advances in organ preservation [84]. However, IRI remains a serious obstacle resulting in graft pancreatitis and possible graft loss [84]. Therefore, the development of novel approaches against IRI in PTx is crucial for further progress.

It is not surprising that with the growing amount of evidence indicating MLT as an effective substance against IRI, it was also tested in an experimental model of IRI in the pancreas [85]. In this study, IRI in the rat pancreas was induced by clamping the gastroduodenal and inferior splenic arteries for one hour, followed by reperfusion. IRI induced a significantly decreased level of antioxidants (superoxide dismutase, catalase, glutathione peroxidase, and reduced glutathione) and an increase in lipid peroxides (malondialdehyde and 4-hydroxynonenal) in the pancreatic tissue. Histological analysis showed that IRI induced acute pancreatitis, accumulation of inflammatory infiltrates, disruption of tissue structure, cell necrosis, and haemorrhage. MLT ameliorated or prevented changes in oxidative stress, biochemical and histological signs of apoptosis and necrosis, and also restored the glandular function of the organ. Moreover, in 80% of the animals treated with MLT, there were no histological signs of pancreatitis. The authors concluded that preventive or therapeutic administration of MLT protects against the induction of oxidative stress and tissue injury, thereby improving pancreatic function.

Later, promising results of MLT usage against IRI in the pancreas were tested in an experimental PTx model in pigs [63]. This study compared MLT and ascorbic acid (AA) and their influence on the survival of a pancreas graft after transplantation without immunosuppression. MLT significantly delayed acute rejection and prolonged allograft survival. Furthermore, reduced levels of oxidative stress in pancreatic tissue samples during organ harvest, cold ischemia, and reperfusion period were detected. MLT also reduced serum levels of pig-major acute-phase protein/inter-α-trypsin inhibitor heavy chain 4 (pMAP/ITIH_4_) in the early post-transplantation period. Porcine pMAP/ITIH_4_ is an IL6 dependent acute-phase protein whose concentration increases by up to 30-fold when pigs suffer from inflammation or surgical trauma [86]. In comparison, AA did not show such a beneficial effect against IRI after PTx. The authors concluded that MLT may be a useful therapeutic tool for PTx.

In conclusion, MLT was shown to protect the pancreas against IRI in non-transplant settings and in well-designed large animal models of PTx. On the other hand, it remains unclear if MLT is effective only when administered to the donor, the recipient or added to the preservation solutions, or if only some of these steps are enough to achieve a beneficial effect.

### 2.6. Potential Limitations for Basic Research Knowledge Translation to Clinical Practice

Various experimental studies with a focus on the potential role of MLT in experimental transplantation have been performed; however, these studies have several limitations. First, typically young and healthy animals have been used in the preclinical models while patients are older and usually suffer from a variety of comorbidities [87]. Second, the time point of MLT application used in the experimental setting cannot be applied in the clinical setting. In patients, MLT could be given at the earliest after brain death provided that MLT is only indicated for graft protection. In contrast, MLT was administered to the donor before the ischemic period, and discussions regarding the etchical aspects of such an approach in human settings need to be solved [88]. Experimental studies did not exclude the potential threat that MLT might interact with multiple drugs used in human transplantation. Furthermore, most of the studies focused on short-term results, and not on the long-term impact of MLT. Finally, animal studies tend to overestimate the novel treatment potential because negative or uncertain results are often unpublished. 

## 3. Conclusions

The diversity of settings in which MLT was applied in the field of experimental transplantation is very broad, ranging from a supplement of a perfusion solution to a potential immunosuppressant. However, most of the experimental studies focused on the anti-IRI properties of MLT. Generally, those studies could be divided into two types, including studies investigating MLT as a supplement to preservation solutions on the one hand, and studies investigating MLT as a drug against IRI applied orally or parenterally on the other hand. This review showed that addition to the preservation solution always led to positive results while studies focusing on the prevention of IRI gave controversial results. Moreover, the beneficial effect of MLT added to preservation solutions was demonstrated in both small and large animal studies. Therefore, we are convinced that the data accumulated to date are sufficient to investigate MLT as an additive to preservation solutions targeting IRI in first clinical trials. According to the data obtained from experimental studies, it seems that the adequate concentration would be 100 µmol/L. However, the optimal concentration should be determined in early-phase clinical trials. Other MLT properties, such as immunosuppression or the promotion of organ regeneration, seem to be promising but should be investigated more deeply in experimental models prior to clinical trials. Another objective for future experimental models should be to investigate the capacity of MLT to improve mitochondrial function during IRI.

## Figures and Tables

**Table 1:** Melatonin application in experimental transplantation studies.

Author	Organ	Experi-Mental Animal	Intervention	MLT Application Rout and Dose	Beneficial Effect of Melatonin
Sihai et al. [48]	Heart	Rat	Isolated heart perfusion model after 12 h of static cold storage in St. Thomas solution with or without MLT	Supplement to preservation solution (100 µmol/L)	Increased functional parameters (aortic flow, cardiac output, systolic pressure, left ventricle minute work and coronary flow) Decreased creatine kinase release Better preservation of high-energy phosphates in heart tissue (phosphocreatine and β-adenosine triphosphate) Blunted histological injury signs in electron microscopy
Jung et al. [33]	Heart	Rat	Heart transplantation	Different groups received a low dose (20 mg/kg) or high dose (200 mg/kg) of MLT orally (via gavage)	High-dose melatonin therapy resulted in: Reduced lymphocyte proliferative capacity Abrogated acute rejection Significantly prolonged allograft survival
Liu et al. [53]	Heart	Rat	Heart transplantation	Different groups received daily treatment with: MLT (200 mg/kg; via gavage); Cyclosporine (20 mg/kg); MLT (50 mg/kg) co-administered with cyclosporine (5 mg/kg)	Daily treatment with melatonin: 4. Significantly prolonged the cardiac graft survival 5. Significantly alleviated the inflammatory response 6. Reduced apoptosis 7. Highest graft survival results were reached when MLT was co-administered with cyclosporine
Inci et al. [54]	Lung	Rat	Single-lung transplantation after 18 hours of cold ischemia	Donor received intraperitoneal injection of 10 mg/kg MLT ten minutes before organ retrieval. Recipient received intraperitoneal injection of 10 mg/kg melatonin 10 min before reperfusion	Reduced lipid peroxidation and myeloperoxidase activity in lung tissue Reduced nitrite levels in bronchoalveolar lavage Improved functional parameters (Higher oxygenation level and reduced peak airway pressure)
Santana-Rodriguez et al. [55]	Lung	Rat	Lung transplantation after 6 hours of ischemia	Recipient received a single dose (10 mg/kg) of MLT applied subcutaneously 50 min before reperfusion	Significant improvement was not observed
Zaouli et al. [56]	Liver	Rat	Isolated steatotic and healthy liver perfusion model after 24 h of static cold storage in IGL-1 solution with or without MLT	Supplement to preservation solution (100 µmol/L)	Lower transaminase levels Higher bile production and BSP clearance Reduced vascular resistance Increased nitrites/nitrates content in the liver also eNOS activity Prevention of oxidative stress and inflammatory cytokine release
Vairetti et al. [57]	Liver	Rat	Isolated healthy liver perfusion model after 20 h of static cold storage in KHB solution with or without MLT	Supplement to preservation solution (200, 100, 50, 25 µmol/L) and supplement to perfusion solution (100 µmol/L)	Increased bile flow production Increased bile bilirubin excretion Increased ATP level preservation in liver tissue
Moussavian et al. [58]	Liver	Rat	Donor preconditioning with multidrug cocktail followed by isolated healthy liver perfusion model after 24 h of static cold storage in HTK solution.	Melatonin (10 mg/kg; intraperitoneally) was included in multidrug cocktail used for donor preconditioning	Abolished inflammation response Prevented parenchyma injury
Heesen et al. [59]	Liver	Rat	Donor preconditioning with multidrug cocktail followed by isolated steatotic liver perfusion model after 24 h of static cold storage in HTK solution.	Melatonin (10 mg/kg; intraperitoneally) was included in multidrug cocktail used for donor preconditioning	Abolished inflammation response Reduced parenchyma injury
Zaouli et al. [60]	Liver	Rat	Isolated steatotic liver perfusion model after 24 h of static cold storage in IGL-1 solution with or without MLT + TMZ cocktail	Supplement to preservation solution (100 µmol/L of MLT combined with 10^−3^ μm of trimetazidine)	Lower transaminase levels Higher bile production and BSP clearance Reduced vascular resistance Reduced hepatic malondialdehyde and glutamate dehydrogenase level Prevention of endoplasmic reticulum stress caused by 5′ AMP-activated protein kinase activation Modulated liver autophagy
Song et al. [61]	Liver	Mice	Hepatic IRI and regeneration associated with partial liver transplantation was investigated in three different models: IRI in partial hepatectomy model IRI in expanded hepatectomy model associated with SFSS SFS liver transplantation model	MLT (10 mg/kg) was administered intraperitoneally before the operation and after reperfusion in IRI and hepatectomy models In SFS, liver transplantation model MLT (20 μg/mL) was used as a supplement for perfusion and storing solution. Additionally, MLT (10 mg/kg) was injected intraperitoneally to the recipient before the transplantation and immediately after the reperfusion	In IRI and hepatectomy models: Reduced liver injury Enhanced liver regeneration Promoted interleukins (IL6, IL10, TNF-α) release from inflammatory (Ly6C+ F4/80+) monocytes. IL6 significantly improved hepatic microcirculation and survival of experimental animals In the SFS liver transplantation model: Promoted graft regeneration Increased recipient survival
Li et al. [62]	Kidney	Rat	Kidney transplantation after 24 h of static cold storage in HTK solution.	Single dose of MLT (50 mg/kg; via gavage) administered to donor 2 h before explantation	Improved survival Decreased blood urea nitrogen, creatinine, transaminases, and lactate dehydrogenase levels in plasma after transplantation Significantly reduced histological index of tubular damage Induced enzymatic activity of superoxide dismutase in kidney tissue Reduced activity of lipid hydroperoxide in kidney tissue Down-regulated expression of NF-κB p65, iNOS, and caspase-3 in kidney tissue
Garcia-Gil et al. [63]	Pancreas	Pig	Pancreas transplantation model without immunosuppression after 8 h of static cold storage in University of Wisconsin solution.	Donor received MLT (10 mg/kg, intravenously) 30 min before vascular clamping. Recipients received MLT (10 mg/kg, intravenously) 30 min before reperfusion and later daily for first 7 postoperative days Pancreas were stored in UW solution supplemented with 5 mM of MLT	Significantly delayed acute rejection Prolonged allograft survival Reduced level of oxidative stress markers (malondialdehyde, 4-hydroxyalkenals) Reduced serum level of pMAP/ITIH_4_ in the early post-transplantation period
